# Resveratrol Inhibits Porcine Intestinal Glucose and Alanine Transport: Potential Roles of Na^+^/K^+^-ATPase Activity, Protein Kinase A, AMP-Activated Protein Kinase and the Association of Selected Nutrient Transport Proteins with Detergent Resistant Membranes

**DOI:** 10.3390/nu10030302

**Published:** 2018-03-03

**Authors:** Stefanie Klinger, Gerhard Breves

**Affiliations:** Department of Physiology, University of Veterinary Medicine Hannover, Foundation, Bischofsholer Damm 15, 30173 Hannover, Germany; gerhard.breves@tiho-hannover.de

**Keywords:** AMPK, intestinal transport, PKA, SGLT1, phosphorylation, Ussing chamber

## Abstract

Background: Beneficial effects of Resveratrol (RSV) have been demonstrated, including effects on transporters and channels. However, little is known about how RSV influences intestinal transport. The aim of this study was to further characterize the effects of RSV on intestinal transport and the respective mechanisms. Methods: Porcine jejunum and ileum were incubated with RSV (300 µM, 30 min) in Ussing chambers (functional studies) and tissue bathes (detection of protein expression, phosphorylation, association with detergent resistant membranes (DRMs)). Results: RSV reduced alanine and glucose-induced short circuit currents (ΔI_sc_) and influenced forskolin-induced ΔI_sc_. The phosphorylation of sodium–glucose-linked transporter 1 (SGLT1), AMP-activated protein kinase (AMPK), protein kinase A substrates (PKA-S) and liver kinase B1 (LKB1) increased but a causative relation to the inhibitory effects could not directly be established. The DRM association of SGLT1, peptide transporter 1 (PEPT1) and (phosphorylated) Na^+^/H^+^-exchanger 3 (NHE3) did not change. Conclusion: RSV influences the intestinal transport of glucose, alanine and chloride and is likely to affect other transport processes. As the effects of protein kinase activation vary between the intestinal localizations, it would appear that increasing cyclic adenosine monophosphate (cAMP) levels are part of the mechanism. Nonetheless, the physiological responses depend on cell type-specific structures.

## 1. Introduction

Resveratrol (RSV) is supposed to mediate beneficial effects on a wide variety of physiological parameters. Besides influences on mitochondrial signaling pathways or anti-inflammatory processes, effects on several ion transporters and channels have been described. Although the bioavailability of RSV is supposed to be low [1] and many studies regarding these effects were carried out using cell culture models, food additives are distributed commercially and efforts are made to enrich rice with RSV [2,3]. Evidence for RSV effects on intestinal chloride secretion [4] and on the expression of the H^+^-coupled peptide transporter 1 (PEPT1) [5,6] have been postulated. In a previous study on the influence of RSV on intestinal glucose absorption, we showed in Ussing chamber studies that short-time incubation with RSV (30 min) decreases the electrogenic Na^+^-dependent glucose transport (measured as change in short circuit currents (I_sc_)) in jejunal and ileal samples of porcine small intestines [7], which is mainly mediated by the sodium–glucose-linked transporter 1 (SGLT1). Long-term regulation of SGLT1 is based on enhanced gene expression, while the apical insertion is involved in short-term regulation [8]. Apart from this, there are different mechanisms that are relevant for regulating the activity of SGLT1.

1. SGLT1 activity is modulated by different intracellular pathways responsible for the functionality of enterocytes. SGLT1 is phosphorylated by protein kinase A (PKA) at Serin 418 (Ser418), which is likely to activate SGLT1, although, depending on the species, inhibitory effects have also been described [9,10]. AMP-activated protein kinase (AMPK) also influences SGLT1. Inhibitory effects [11] were observed as activating responses as well [12,13].
2. The secondary active transport of glucose across the apical membrane depends on the inwardly directed Na^+^ gradient that is maintained by the basolateral Na^+^/K^+^-ATPase. Additionally, membrane potential affects Na^+^-coupled sugar transport and the activity of SGLT1 [14,15]. The local Na^+^-evoked, SGLT1-inhibiting depolarization at the apical membrane is compensated by potassium (K^+^) channels localized in the basolateral or apical enterocyte membrane, depending on paracellular tightness [16,17].
3. The activity of several transmembrane proteins depends on their lipid environment including the membrane fluidity and their association with detergent-resistant membrane (DRM) fractions (or lipid rafts) that represent membrane fractions enriched in cholesterol and sphingolipids. This is also true for the activity of SGLT1 that increases with decreasing membrane fluidity [18] and decreases with decreasing membrane cholesterol content of the membrane [18,19,20].

**Figure 1:**
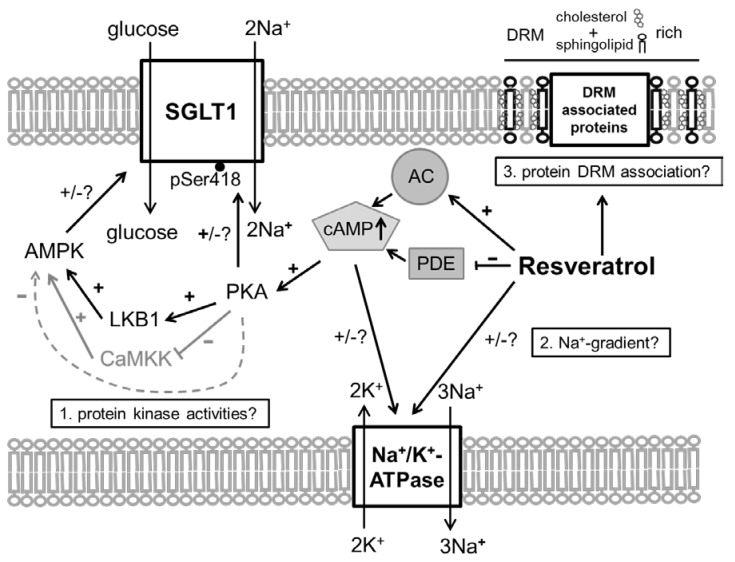
Schematic illustration of how Resveratrol (RSV) may affect intestinal glucose transport. 1. The activation of adenylate cyclase (AC) [22,23] and inhibition of phosphodiesterases (PDE) [21] and the subsequent increased in intracellular cyclic adenosine monophosphate (cAMP) levels are likely to activate protein kinase A (PKA) that is able to phosphorylate sodium–glucose-linked transporter 1 (SGLT1) at Ser418 with species-dependent effects [9,10]. PKA is also able to modulate the activity of the AMP-activated protein kinase (AMPK) via changing the activities of AMPK activating upstream kinases (liver kinase B1 (LKB1) activation [25,26], calcium/calmodulin-dependent protein kinase kinase (CaMKK) inhibition [29]) or by direct phosphorylation of AMPK (inhibition) [28]. 2. Resveratrol may (cAMP-mediated) exert effects on the Na^+^/K^+^-ATPase activity [30,31] and therefore change the driving force for Na^+^-coupled transport processes as the absorption of glucose and alanine. 3. Since Resveratrol is known to affect membrane properties and influences the formation of detergent resistant membrane fractions (DRM) [32,35], the association of nutrient transport proteins with DRMS and therefore their activity may be changed by Resveratrol.

The inhibitory effect of RSV on Na^+^-dependent glucose transport may be associated with all these regulatory pathways or mechanisms (see also Figure 1):

1. RSV acts on intracellular pathways. In this context, the inhibition of phosphodiesterases (PDE) [21], or also adenylate cyclase activation [22,23], leading to increased levels of cyclic adenosine monophosphate (cAMP), is of interest as the main stimulus for PKA activation. Additionally, RSV is known to activate AMPK [21]. Besides mechanisms related to cellular energy status, AMPK is activated by phosphorylation at threonine 172 (Thr172) by upstream kinases including liver kinase B1 (LKB1) [24]. In turn, it has been shown that activating effects of RSV on AMPK require LKB1 and that RSV activates LKB1 [25,26]. The role of PKA in regulating AMPK is complex since the possibility of activating LKB1 and therefore promoting AMPK activity has been described [27] as well as the potential to inhibit AMPK by phosphorylation or by inhibiting the activating AMPK upstream kinase calcium/calmodulin-dependent protein kinase kinase (CaMKK) [28,29].
2. In addition to potential effects of resveratrol on the membrane potential by influencing the K^+^ conductance, RSV may also exert direct effects on the activity of the Na^+^/K^+^-ATPase. In erythrocytes, RSV activates the pump [30], but assuming a cAMP-mediated effect it has to be taken into account that the effects of cAMP on the activity of Na^+^/K^+^-ATPase are tissue selective [31].
3. RSV is known to affect membrane properties by accumulating in the outer leaflet of the lipid bilayer [32] or in DRMs and one way to enter the cell is via raft-dependent endocytosis [33,34]. There is also evidence that RSV influences the formation of lipid rafts [35], which might be associated with the variety of effects that are exhibited by RSV.

The aim of this study was to identify which of these pathways could be responsible for the inhibition of Na^+^-dependent glucose transport, as all of the mentioned pathways are not only able to influence the absorption of glucose, which could be desirable under circumstances as obesity or diabetes, but also the absorption of other nutrients or ions. Porcine intestinal tissues were used because of their suitability as a model for the human small intestines compared to rodent animal models or cell culture models [36,37]. We investigated, with regard to the above-mentioned aspects, whether short-term incubation with RSV:

1. may also lead to an inhibition of other intestinal Na^+^-dependent transport processes such as Na^+^-dependent alanine transport and whether the activity of the Na^+^/K^+^-ATPase is altered;
2. leads to an activation of AMPK, PKA and LKB1 and to changes in the apical expression and phosphorylation of SGLT1 at the PKA phosphorylation site Ser418;
3. changes the distribution of SGLT1 and other putatively DRM-associated transport proteins (Na^+^/H^+^-exchanger 3 (NHE3) and its phosphorylated forms and PEPT1) between DRM and non-DRM fractions in the apical membrane of enterocytes.

## 2. Materials and Methods

### 2.1. Animals and Tissue Removal

In total, tissues from ten weaned piglets (Sus scrofa domestica, German Landrace × Large White, body weight 31 ± 3 kg) were used. All animals received care according to the German Animal Protection Law. The pigs were kept on a conventional fattening diet (feeding twice daily) and had access to drinking water ad libitum for at least ten days.

On the day of slaughter, the morning feed was offered two hours prior to slaughter. The pigs were slaughtered by stunning with subsequent carotid artery bleeding. Intestinal segments were removed within 15 min after stunning. Chymus was removed with cold saline (4 °C). Tissues were stored in serosal buffer solution (see below) for Ussing chamber experiments that were started within 30 min.

According to the German Animal Protection Law (§7), this procedure (slaughter of animals and tissue removal for scientific purposes) is not classified as an animal experiment and has not to be approved by the Animal Care and Use Committee but has to be announced to the university’s animal welfare officer, which was done on 28 August 2015.

### 2.2. Ussing Chamber Experiments

The third meter distal to the pylorus (jejunum) and the first meter proximal to the ileocaecal valve (ileum, first 30 cm discarded) were used for Ussing chamber experiments. The mucosa was separated from muscle layers and incubated in standard Ussing chambers (exposed serosal area 1.13 cm^2^). In the Ussing chamber system, tissues are fixed between the two halves of an Ussing chamber serving as a barrier to form a mucosal and a serosal compartment that are filled with appropriate buffer solutions (see below). Using a computer-controlled voltage clamp device (Mussler Scientific Instruments, Aachen, Germany), the transepithelial potential difference (PD_t_), the tissue conductance (G_t_) and short circuit current (I_sc_) were measured. All experiments were carried out under in the short circuit mode (application of a direct current setting PD_t_ to zero). Under these conditions, changes in I_sc_ (ΔI_sc_) represent changes in the transepithelial net ion transfer. The mucosal to serosal movement (absorption) of cations such as Na^+^ as well as the serosal to mucosal movement (secretion) of anions such as Cl^−^ result in an increase in I_sc_.

**Figure 2:**
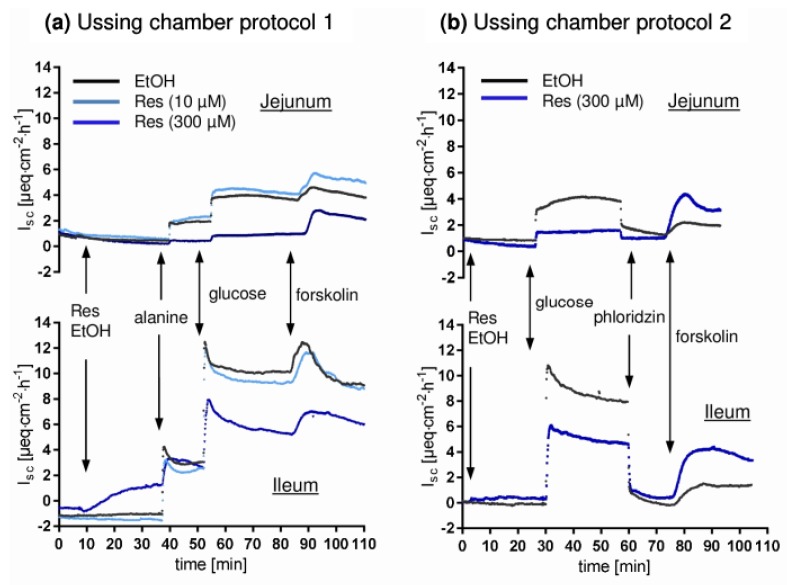
Representative time course of short circuit currents (I_sc_) measured in Ussing chamber experiments. An increase in I_sc_ represents either transepithelial cation absorption or anion secretion. (**a**) protocol 1 (**b**) protocol 2. The black line represents one solvent control chamber while light blue and dark blue represent the low (10 µM) and high (300 µM) concentrations of RSV. Glucose and alanine were added mucosal (10 mM), while mannitol was added to the serosal compartment for osmotic reasons. Phlorizin (mucosal) and forskolin (serosal) were used at a final concentration of 0.01 mM.

Buffer solutions were continuously aerated with carbogen and the temperature was kept at 39 °C. All mucosal additions exceeding 1 mM were osmotically balanced by the serosal addition of mannitol. Two Ussing chamber protocols were applied, using five animals per protocol. Exemplary time courses are shown in Figure 2. The buffer solutions consisted of (mM): mucosal 113.6 NaCl, 5.4 KCl, 0.2 HCl, 1.2 MgCl_2_, 1.2 CaCl_2_, 21 NaHCO_3_, 1.5 Na_2_HPO_4_, 2.0 mannitol, 20 HEPES (4-(2-hydroxyethyl)-1-piperazineethanesulfonic acid ), 0.01 indomethacin; serosal: 113.6 NaCl, 5.4 KCl, 0.2 HCl, 1.2 MgCl_2_, 1.2 CaCl_2_, 21 NaHCO_3_, 1.5 Na_2_HPO_4_, 2.0 mannitol, 7.0 HEPES, 10 Glucose, 6.0 Na-gluconate, 0.01 indomethacin. NaCl, KCl, HCl, MgCl_2_, CaCl_2_, NaHCO_3_, Na_2_HPO_4_, glucose and Na-gluconate were obtained from Merck KGaA, Darmstadt, Germany. Mannitol and indomethacin were obtained from Sigma-Aldrich, St. Louis, MO, USA while HEPES was obtained from Carl Roth GmbH + Co.KG, Karlsruhe, Germany.

Details protocol 1: After equilibration (20 min), tissues were incubated with RSV (trans-Resveratrol, Sigma-Aldrich, St. Louis, MO, USA), dissolved in ethanol (10 µM or 300 µM mucosal) or ethanol as solvent control (here and in all other experiments: 20 µL per 10 mL buffer solution, 0.2% *v*/*v*). After 30 min, alanine (10 mM, mucosal) was added followed by the addition of glucose (10 mM mucosal, 15 min after alanine). Details protocol 2: After equilibration (20 min), 300 µM RSV (mucosal) and the AMPK activators D942 (40 µM mucosal and serosal, Cayman Chemical Company, Arbor, MI, USA) and metformin (1 mM mucosal, Merck KGaA, Darmstadt, Germany) or the respective solvent (ethanol, DMSO, H_2_O) were added. After 30 min, glucose (10 mM mucosal) was added and after another 30 min, phlorizin (0.5 mM mucosal, Sigma Aldrich) was applied. At the end of both protocols, forskolin (10 µM serosal, Sigma-Aldrich) was added, causing an increase in I_sc_ due to cAMP-induced chloride secretion.

### 2.3. Tissue Incubations for Expression Analysis

For Western blot analysis, intestinal segments of 20 cm (second meter distal to the pylorus (jejunum) and the second meter proximal to the ileocaecal valve (ileum)) were incubated in tissue baths designed to create a mucosal and a serosal compartment. This enables the use of different buffer solutions in both compartments according to the buffers used in Ussing chamber studies. After tissue resection, tissues were stored in cold serosal buffer solution. Within 40 min after tissue removal, tissues were transferred to the baths with warm (39 °C), gassed serosal buffer in the serosal compartment and mucosal buffer that already contained the substance of interest or the respective solvent. Again, two slightly different incubation protocols were used. In protocol 1, RSV was used in two concentrations (10 µM and 300 µM mucosal). In protocol 2, 300 µM RSV (300 µM mucosal) and metformin (1 mM mucosal) were used. D942 (40 µM) was used on both, the mucosal and the serosal side. Therefore, only the mucosa was incubated in small tubes containing the serosal buffer solution. Incubations were carried out for 30 min as in Ussing chamber experiments. Afterwards, the tissues were frozen in liquid nitrogen and stored at −80 °C for tissue preparations.

### 2.4. Tissue Preparations

During the preparations, samples and buffer solutions were kept cold and protease and phosphatase inhibitor cocktails (P8340/P5726; Sigma-Aldrich, St. Louis, MO, USA) were added at the end of the preparation. No phosphatase inhibitors were used for the preparation of homogenates because no Na^+^/K^+^-ATPase is measurable when using phosphatase inhibitors.

Cell lysates were obtained after homogenization (Potter-Elvehjem homogenisator) of 1 g mucosa in 3 mL lysis buffer (50 mM TRIS (tris (hydroxymethyl) aminomethane, Sigma-Aldrich, St. Louis, MO, USA) , 150 mM NaCl, 2 mM EDTA (ethylenediaminetetraacetic acid, Sigma-Aldrich, St. Louis, MO, USA), 1% Nonidet P40 substitute (Fluka, Buchs Switzerland, now Sigma-Aldrich, St. Louis, MO, USA), 0.5 % *w*/*v* Na-deoxycholate Fluka, Buchs Switzerland, now Sigma-Aldrich, St. Louis, MO, USA, 0.1% *w*/*v* SDS (sodium dodecyl sulfate, Sigma-Aldrich, St. Louis, MO, USA), incubation (1 h, 4 °C in a shaker) and centrifugation (10,000× *g*, 10 min, 4 °C). Apical enterocyte membranes were enriched as previously described [38]. Cell homogenates for measuring the activity of Na^+^/K^+^-ATPase were obtained as supernatant after homogenization (2 mM TRIS, 50 mM mannitol, pH 7.1, 9 mL/g tissue) and centrifugation (2000 g, 15 min, 4 °C). Protein contents were measured using Bradford assay.

Apical enterocyte membranes were also used for the enrichment of apical DRMs using the FocusTM Global Fractionation Kit (G-Bioscience, St Louis, MO, USA) for investigating protein association with DRM. 1.2 mg total protein was subjected to the DRM preparation procedure recommended by the manufacturer. As DRM preparation resulted in low protein content and since the detergents disturbed the measurement of protein concentrations, equal volumes were used for Western blot. For verifying DRM enrichment, Na^+^/K^+^-ATPase was used as a non-DRM marker and flotillin-1 was used as a DRM marker.

### 2.5. Western Blot

Proteins were separated via SDS-PAGE and blotted on nitrocellulose membranes (tank blot, blotting buffer: 25 mM TRIS base, 192 mM glycine, 20% (*v*/*v*) methanol, 100 V, 90 min). Membranes were blocked (60 min, RT, 5% non-fat dry milk in TBST). For detailed information on the amount of protein loaded, denaturing conditions and antibodies for SGLT1, pSGLT1, pAMPK, AMPK and phosphorylated PKA substrates (PKA-S) used see [38]. LKB1 (3047, cell signaling) and pLKB1 Ser428 (3482, Cell Signaling Technology, Cambridge, UK) were detected using the same protocol as for pAMPK/AMPK.

Additionally, the following primary antibodies and conditions were used: NHE3: denaturing at RT for 20 min, primary antibodies: rabbit-anti-NHE3 (sc-16103-R, Santa Cruz Biotechnology, Dallas, TX, USA), mouse-anti-NHE3 Ser552 (NB110-81529, Novus Biologicals, Littleton, CO, USA, mouse-anti-NHE3 Ser605 (NB110-74678SS, Novus Biologicals); PEPT1, Na^+^/K^+^-ATPase, flotillin-1: denaturing at 95 °C for 5 min, goat-anti-PEPT1 (19917, Santa Cruz), mouse-anti-Na^+^/K^+^-ATPase (ALX-804-082, Enzo life Sciences, New York, NY, USA), rabbit-anti-flotillin-1 (3253, Cell Signaling). Depending on the origin of the primary antibody, the following secondary antibodies were used: goat-anti-rabbit HRP (7074, Cell Signaling for cell signaling primary antibodies, A9169, Sigma-Aldrich for others), goat-anti-mouse (A2304, Sigma-Aldrich), rabbit-anti-goat (A5429, Sigma-Aldrich).

For determining the phosphorylation levels, both primary antibodies were incubated with the same membrane. After blotting and blocking, the phosphospecific antibody was used. Afterwards, the antibody was removed by incubating the membrane with stripping buffer (0.2 M Glycin, 0.05% Tween 20, 1% SDS, 45 min, RT), washed, blocked again and incubated with the respective non phosphospecific primary antibody. Total protein was stained with Indian ink [38] or, if DRMs were blotted, with the Pierce™ Reversible Protein Stain Kit for Nitrocellulose Membranes (Thermo Fisher Scientific, Waltham, MA, USA).Chemiluminescence signals were detected using SuperSignal^®^ West Dura Extended Duration Substrate or SuperSignal^®^ West Femto Maximum Sensitivity Substrate (Thermo Fisher Scientific) and the ChemiDoc™ MP imaging system with the Image Lab 5.2 software (Bio-Rad Laboratories, Hercules, CA, USA) for band analysis.

### 2.6. Data Analysis and Statistics

Data are given as means ± standard deviation (MW ± SD). For calculating ΔI_sc_, the maximal changes after adding the respective substance were used. Although two experimental protocols were used, parameters that were measured in both protocols were analyzed together (*n* = 10) since there were no differences between the experimental series. Parameters unique for one protocol (*n* = 5) were the DRM association and some ΔI_sc_ measurements (alanine, D942, metformin). Statistical analysis for Ussing chamber results was performed using paired students-test when a Gaussian distribution was given. Otherwise, Wilcoxon matched-pairs signed rank test was used. The protein phosphorylation levels were determined by calculating the phosphorylation levels for each sample by dividing the intensity of the phosphospecific band by the intensity of the non-phosphospecific band. The effect of RSV on phosphorylation levels was calculated by dividing the phosphorylation levels for the treated samples by the respective control sample. The means of those relative changes were tested for significance using one sample t-tests with the hypothetical values set at 1.

## 3. Results and Discussion

Due to the fact that the low concentration of RSV (10 µM) did not show any effects in Ussing chamber experiments (Figure 2), the respective data are not shown. The corresponding samples were not included in further experiments regarding protein expression and DRM association. All effects described below refer to 300 µM RSV (mucosal).

**Figure 3:**
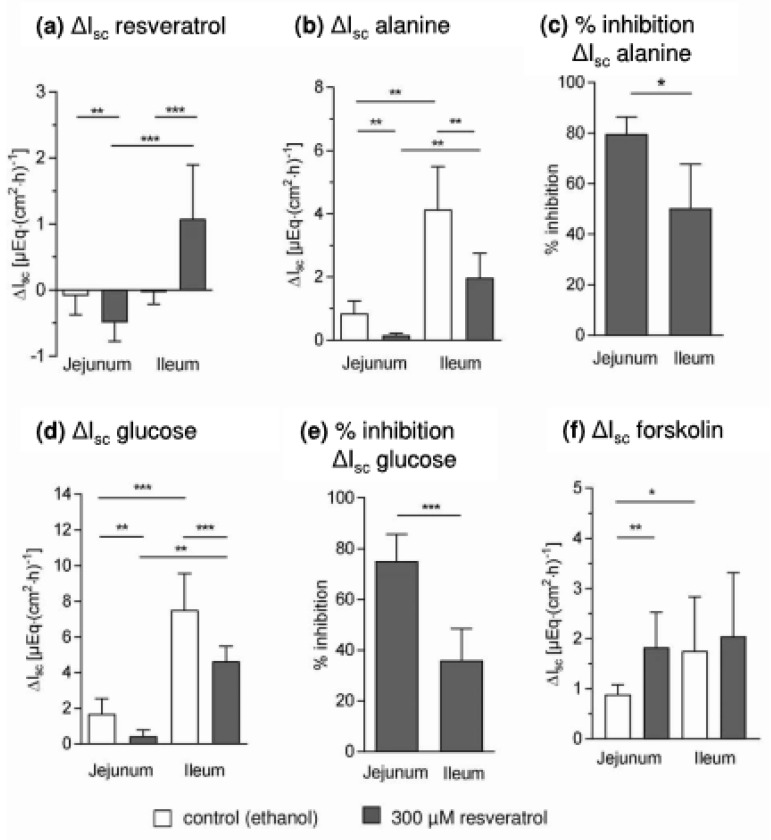
Effects of resveratrol on short circuit currents (I_sc_) and respective changes (ΔI_sc_) measured in Ussing chamber experiments with jejunal and ileal mucosae (**a**) ΔI_sc_ after the mucosal addition and 30 min of incubation with resveratrol (300 µM) or solvent (20 µL ethanol) (**b**) alanine-induced ΔI_sc_ (10 mM, mucosal) (**c**) percentage inhibition in ΔI_sc_ in relation to control chambers after the addition of alanine (**d**) glucose-induced ΔI_sc_ (10 mM, mucosal) (**e**) percentage inhibition in ΔI_sc_ in relation to control chambers after the addition of glucose (**f**) forskolin-induced ΔI_sc_ (0.01 mM, serosal). MW ± SD, *n* = 10 apart from (**b**,**c**) were *n* = 5, * *p* ≤ 0.05, ** *p* ≤ 0.01, *** *p* ≤ 0.001.

I_sc_ changed after adding RSV (Figure 3a). After 30 min, there was a significant decrease in the jejunum (−0.49 ± 0.28 µE∙(cm^2^∙h)^−1^, *p* = 0.002, *n* = 10), while an increase was measured in ileal samples (1.08 ± 0.82 µE∙(cm^2^∙h)^−1^, *p* = 0.0008, *n* = 10). Only in ileal tissues was a minor effect of RSV on ΔG_t_ observed (control: 1.23 ± 0.89 mS∙cm^−2^, RSV 0.04 ± 1.61 mS∙cm^−2^, *p* = 0.018, *n* = 10). This indicates that RSV changes the ion conductance in the absence of glucose and under these conditions influences on chloride secretion have to be considered [4]. RSV-induced chloride secretion would explain the increase in I_sc_ in ileal samples, which will be the subject of further studies. If the decreased I_sc_ in the jejunum is only based on changes in chloride secretion this would indicate an inhibitory instead of an activating action of resveratrol on chloride secretion in this intestinal segment or the involvement of other ion currents, which is also conceivable when considering the multiple effects of RSV.

The forskolin-induced ΔI_sc_ (Figure 3f) differed between the intestinal segments under control conditions (jejunum: 0.89 ± 0.19 µE∙(cm^2^∙h)^−1^, ileum: 1.76 ± 1.08 µE∙(cm^2^∙h)^−1^, *p* = 0.0273). A RSV-induced increase to 1.8 ± 0.70 µE∙(cm^2^∙h)^−1^) was observed for jejunal samples (*p* = 0.002), whereas RSV did not affect the ileal forskolin-induced ΔI_sc_. It has to be elucidated in further experiments whether the mechanisms underlying this effect and the effects of RSV on basal I_sc_ are associated.

The alanine-induced jejunal ΔI_sc_ (0.85 ± 0.39 µE∙(cm^2^∙h)^−1^) was reduced to 0.16 ± 0.06 µE∙(cm^2^∙h)^−1^ (*p* = 0.0097, *n* = 5) in chambers incubated with RSV (Figure 2b), which is equivalent to an inhibition of 79.6 ± 7% (Figure 3c). The ileal ΔI_sc_ after adding alanine (4.13 ± 1.38 µE∙(cm^2^∙h)^−1^) was higher than the jejunal ΔI_sc_ (*p* = 0.0067, *n* = 5) and was reduced to 1.97 ± 0.78 µE∙(cm^2^∙h)^−1^ by RSV (Figure 3b). The percentage of inhibition (50.3 ± 17.5%) was lower than in the jejunum (*p* = 0.012, *n* = 5, Figure 3c).

Almost the same pattern of effects was observed for ΔI_sc_ measured after adding glucose (Figure 3d,e). As already shown [7], the maximal jejunal ΔI_sc_ was decreased by RSV from 1.68 ± 0.85 µE∙(cm^2^∙h)^−1^ to 0.44 ± 0.36 µE∙(cm^2^∙h)^−1^ (*p* = 0.002, *n* = 10). The maximal ileal ΔI_sc_ (7.82 ± 2.05 µE∙(cm^2^∙h)^−1^) was higher (*p* < 0.0001, *n* = 10) and decreased to 4.47 ± 0.97 µE∙(cm^2^∙h)^−1^ after RSV preincubation (*p* = 0.0003, *n* = 10). The relative jejunal inhibition (75.0 ± 10.7 %) was again more pronounced than the relative inhibition in ileal samples (35.9 ± 12.6 %, *p* = 0.0001, *n* = 10, Figure 3e).

The SGLT1 inhibitor phlorizin decreased the I_sc_ to levels observed before the addition of glucose in ileal samples whereas the jejunal I_sc_ remained slightly higher than the initial values, which was more pronounced in RSV-treated chambers (differences between initial I_sc_ and I_sc_ after phlorizin: control: 0.22 ± 0.13 µE∙(cm^2^∙h)^−1^, RSV: 0.59 ± 0.35 µE∙(cm^2^∙h)^−1^, *p* = 0.0439). The effect of RSV on alanine- and glucose-induced ΔI_sc_ were in close correlation (*R*^2^ = 0.84).

This indicates that the known effects of RSV on glucose transport are not restricted to a specific inhibition of SGLT1. It has to be discussed that the effects of RSV could be mediated by more principle effects on cellular processes. Increased cAMP levels due to RSV-induced PDE inhibition [21] may be a general mechanism by which RSV alters the enterocytes transport properties.

**Figure 4:**
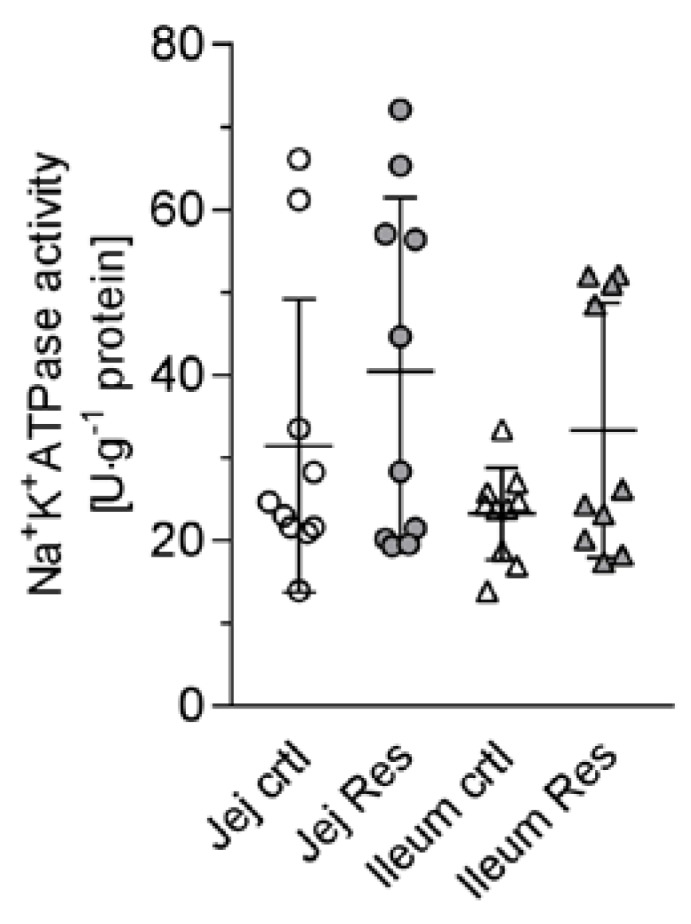
Activity of Na^+^/K^+^-ATPase in jejunal and ileal control and resveratrol-treated samples as measured in tissue homogenates. No significant changes after resveratrol incubation were observed (paired t-test, MW ± SD, *n* = 10).

One aspect may be based on influencing the activity of Na^+^/K^+^-ATPase, and therefore the inwardly directed Na^+^ gradient, as it is known that the Na^+^/K^+^-ATPase activity is influenced by cAMP levels [31]. As shown in Figure 4, the activity of Na^+^/K^+^-ATPase did not change in RSV-treated tissues. In some samples the activity was even increased in a similar manner to that which has been observed for erythrocytes [30].

**Figure 5:**
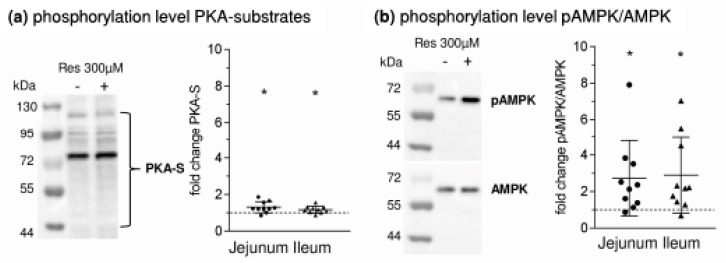

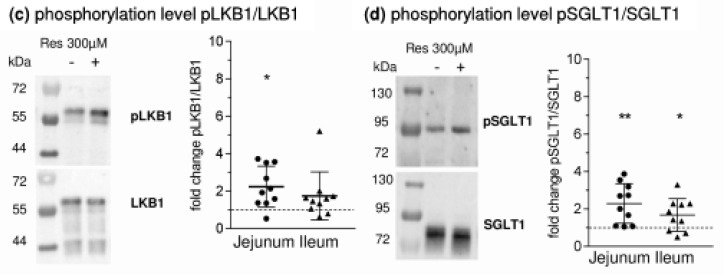
Changes in the phosphorylation levels of (**a**) PKA substrates (**b**) AMPK (**c**) LKB1 (**d**) SGLT1 after incubation with Resveratrol (300 µM, mucosal). One exemplary Western blot is shown together with the relative changes when setting the phosphorylation levels of control samples at 1. MW ± SD (*n* = 10), tested against 1 using one sample t-tests. * *p* ≤ 0.05, ** *p* ≤ 0.01.

The elevation of intracellular cAMP levels has numerous and interacting effects on the activity of many intracellular pathways with a variety of potential effects on cellular function. Changes in intracellular cAMP concentrations after incubation with RSV were not measured in the present study. Nevertheless, there is strong evidence that they were elevated as the amount of phosphorylated PKA substrates in apical membrane increased after RSV incubation (jejunum: 1.30 ± 0.30 fold; *p* = 0.012, ileum: 1.17 ± 0.19 fold; *p* = 0.025, Figure 5a). Also, an AMPK activation was observed as expected since the pAMPK/AMPK ratio measured in cell lysates increased significantly after incubation with RSV (Figure 5b, jejunum: 2.73 ± 2.06 fold; *p* = 0.027, ileum 2.89 ± 2.08 fold; *p* = 0.018). No difference for pAMPK/AMPK was obvious when using apical membrane preparations and no difference in PKA-S was measured in cell lysates (data not shown). pLKB1/LKB1 (Figure 5c) in cell lysates also increased after RSV incubation, but only the jejunal increase significantly differed from 1 (2.24 ± 1.10, *p* = 0.006). Significance was not reached for ileal samples (1.74 ± 1.29, *p* = 0.102) but this was due to the very high increase in one sample.

The amount total SGLT1 did not change after RSV incubation (data not shown), thus genomic responses can be ruled out. The pSGLT1/SGLT1 ratio measured using apical membrane preparations increased after tissue incubation with RSV in both jejunal (2.29 ± 1.06 fold; *p* = 0.004) and ileal (1.67 ± 0.88 fold; *p* = 0.038) samples (Figure 5d).

**Figure 6:**
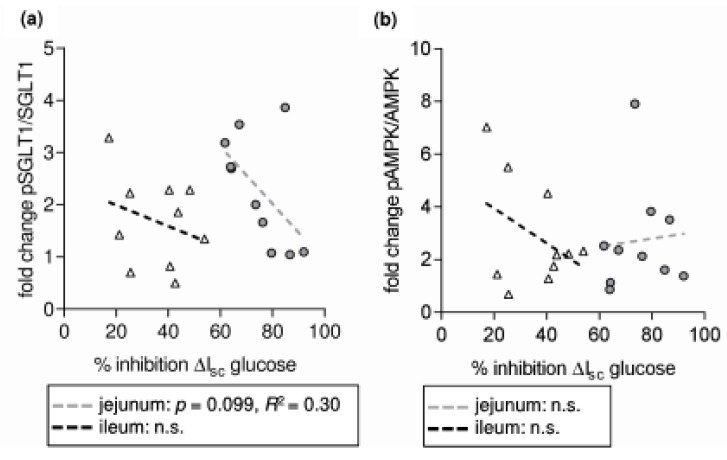

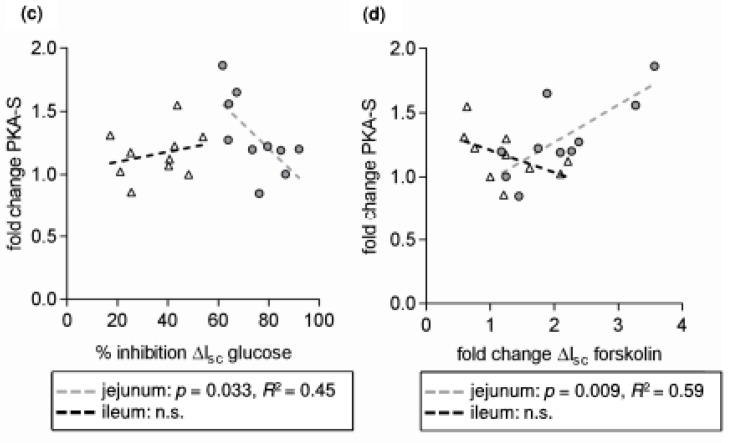
Correlation analysis of the relative changes in protein phosphorylation levels expressed as fold change of resveratrol-treated samples compared to control samples and results from Ussing chamber studies (**a**) fold change in pSGLT1/SGLT1 vs. percent inhibition of glucose-induced ΔI_sc_ (**b**) fold change in pAMPK/AMPK vs. percent inhibition of glucose-induced ΔI_sc_ (**c**) percent inhibition of glucose-induced ΔI_sc_ vs. fold change in the amount of phosphorylated PKA substrates (PKA-S) (**d**) fold change in forskolin-induced ΔI_sc_ vs. fold change in PKA-S. *n* = 10.

Considering the decreased glucose-induced I_sc_ and the increase in pSGLT1/SGLT1, it may be concluded the phosphorylation of SGLT1 has rather an inhibitory impact than an activating one. However, from Figure 6a it seems quite likely that the increase in SGLT1 phosphorylation was activating as was also discussed recently [38] since at least in jejunal samples, the percentage of RSV-induced I_sc_ inhibition decreases with increasing SGLT1 phosphorylation. It can only be speculated whether this may be due to the fact that the inhibitory potential of RSV is lower in this segment and the basal and remaining glucose influx are much higher than in the jejunum.

**Figure 7:**
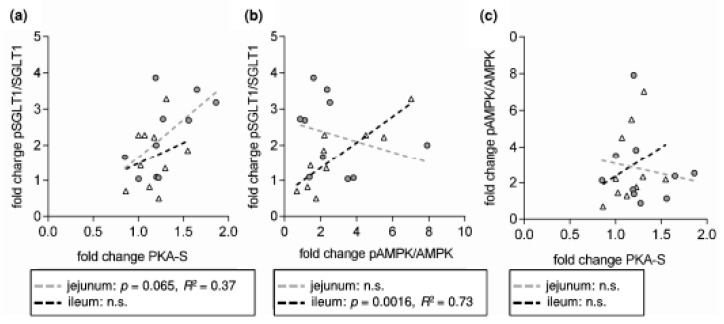
Correlation analysis of the relative changes in protein phosphorylation levels expressed as fold change of resveratrol-treated samples compared to control samples. (**a**) fold change in pSGLT1/SGLT1 vs. fold change in the amount of phosphorylated PKA substrates (PKA-S) (**b**) fold change in pSGLT1/SGLT1 vs. fold change in pAMPK/AMPK (**c**) fold change in PKA-S vs. fold change in pAMPK/AMPK. *n* = 10.

The RSV-induced change in pSGLT1/SGLT1 in the jejunum seems to be attributable to changes in PKA activity since there was a significant correlation between the changes in PKA-S and pSGLT1/SGLT1 (Figure 7a) and, as observed for pSGLT1/SGLT1, a significant negative correlation of the change in PKA-S and the inhibitory potential of RSV on glucose-induced ΔI_sc_ (Figure 6c). Although the mean change in PKA-S was also significant for ileal samples, these correlations were not observed for this intestinal segment. In turn, there was a highly significant positive correlation for the RSV-induced changes of pAMPK/AMPK and pSGLT1/SGLT1 in the ileum (Figure 7c) but this correlation was not accompanied by a respective negative correlation of pAMPK/AMPK with Ussing data (Figure 6b) as was the case for PKA-S in the jejunum (Figure 6c).

It can be summarized that RSV increases the activity of all three kinases. However, this has a different impact on glucose transport, the inhibitory potential of RSV and the phosphorylation level of SGLT1. Regardless, these effects are not the direct reason for the RSV-induced changes in intestinal transport but rather compensate for this inhibition at least in the jejunum.

This is supported by the result that the metformin-induced increase in pAMPK/AMPK (1.30 ± 0.15, *p* = 0.013) and PKA-S (1.31 ± 0.19, *p* = 0.02) in ileal samples did not affect the glucose-induced ΔI_sc_. In rats, a rapid AMPK-mediated decreasing effect of metformin on glucose-induced ΔI_sc_ has been described [11], which is in contrast to the data in the present study. The AMPK activator D942 had no effect on any of the measured parameters apart from a slight increase in the basal ΔI_sc_ for ileal tissues (control: −0.12 ± 0.09 µE∙(cm^2^∙h)^−1^, RSV: 0.45 ± 0.21 µE∙(cm^2^∙h)^−1^, *p* = 0.004, *n* = 5). To our knowledge, this is the first study that used D942 in Ussing chamber experiments and, therefore, its effectiveness cannot be compared with other data.

**Figure 8:**
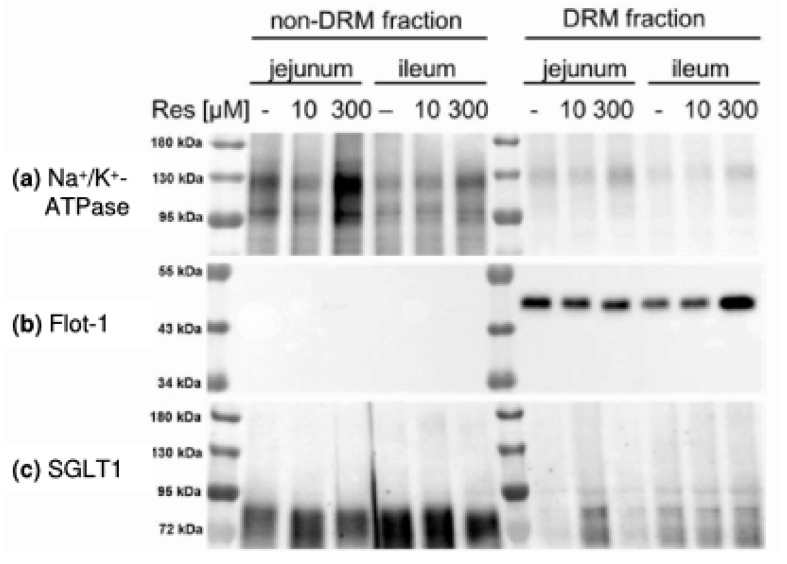

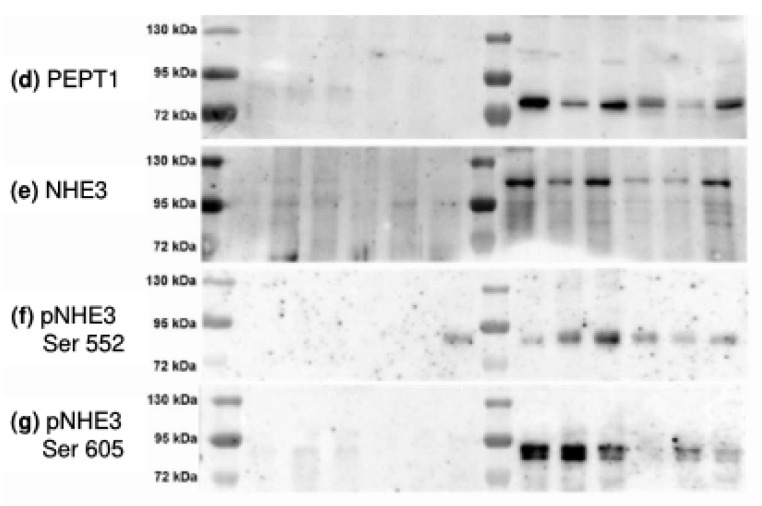
Representative Western blots of (**a**) the non-DRM marker Na^+^/K^+^-ATPase (**b**) the DRM marker flotillin-1 (Flot-1) and the transport proteins SGLT1 (**c**), PEPT1 (**d**), NHE3 (**e**) and phosphorylated NHE3 at Serin522 (**f**) and Serin605 (**g**) in non-DRM and DRM fractions prepared from jejunal or ileal tissues after treatment with resveratrol (10 µM or 300 µM, 30 min).

Nevertheless, it is of importance to discuss why RSV-induced PKA activation had an impact on glucose transport characteristics and also on forskolin-induced chloride secretion (Figure 6d) in jejunal but not in ileal tissues. In this context the compartmentalization of cAMP signaling and PKA anchoring may play a role since in the present study effects of RSV on PKA activation were only observed when apical membrane preparations were used. The subcellular distribution of the membrane associated form of PKA (PKAII) and therefore the compartmentalization of cAMP signaling is regulated by the protein group of PKA anchoring proteins (AKAPs), as for example ezrin [39], resulting in only regional changes in the cAMP concentration in proximity to different proteins including adenylate cyclase [40]. Different PKA/AKAP complexes may be the reason for the different effects of PKA activation in the present study. It has been shown that ezrin is involved in the intestinal glucose-mediated increase in Na^+^ absorption via NHE3 [41]. The PKA association with different AKAPs also results in changes in the transport of calcium (Ca^2+^) or K^+^ for example in cardiomyocytes [42,43]. Both Ca^2+^ and K^+^ are essential for intestinal transport with regard to intracellular signaling and the maintenance of the membrane potential and changes in their transport may explain both the inhibition of glucose and alanine transport as well as the diverse effects of resveratrol on intracellular kinases in the different intestinal segments observed in the present study. Effects of RSV on Ca^2+^ channels and signaling [44,45] and K^+^ channels [46,47] have already been described in several studies. One link between the formation of AKAP/PKA signaling complexes and RSV may be the association of AKAPs and adenylate cyclase with lipid rafts [40,48] and the ability of RSV to influence the properties of bio membranes including lipid raft formation [33,34,35]. We investigated whether RSV changes the association of intestinal nutrient transporters with DRMs. The separation of non-DRM and DRM fractions was verified by detecting marker proteins. While the non-DRM marker Na^+^/K^+^-ATPase was mostly abundant in the non-DRM fraction (Figure 8a), the DRM marker flotillin-1 was solely detectable in the DRM fraction (Figure 7b). Except SGLT1 (Figure 8c), all transport proteins investigated were mostly found in the DRM fraction (Figure 8d–g).

Incubation with RSV did not significantly change the distribution of any protein within the DRM fraction (Table 1) but we had to accept high standard deviations. Therefore, further experiments should be carried out in order to verify this finding and the DRM association of other proteins like AKAPs and adenylate cyclase should be included.

## 4. Conclusions

With regard to the objectives of this study, it can be summarized that Na^+^-dependent alanine transport is inhibited by RSV and that the segment-specific characteristics are similar to those observed for the RSV-induced inhibition of Na^+^-dependent glucose transport, while no alterations in the activity of Na^+^/K^+^-ATPase were observed. AMPK, PKA and LKB1 were activated to different extents (AMPK > LKB1 > PKA) and with different, also segment-specific, impacts on the changes in I_sc_ and SGLT1 phosphorylation. The results indicate that the effect of RSV on the phosphorylation level of SGLT1 is not the cause for the inhibition of Na^+^-dependent glucose transport but rather a consequence thereof that even partially counteracts the inhibitory effect of RSV. As also the basal I_sc_ and the forskolin-induced chloride secretion were affected and since these effects were different in the intestinal segments, it can be concluded that effects of RSV are not simply based on influencing the intracellular cAMP levels. Rather, these may be based on complex influences with regard to structures that are essential for the regulation and specialization of cellular functions, as for example, the compartmentalization of cAMP-signaling and PKA anchoring, which is also supported by the fact that no correlation between the change in pAMPK/AMPK and PKA-S was found. Assuming such a general mechanism of RSV, it is likely that other intestinal nutrient and ion transport systems are also impaired by RSV. It is therefore of importance to identify them as well as the mechanisms that are responsible for the observed effects in more detail.

## Figures and Tables

**Table 1:** Association of transport proteins with the DRM fraction prepared from jejunal and ileal apical membranes after incubation with resveratrol (300 µM, 30 min) expressed as changes (means ± SD) in the protein/flotillin-1 ratio. The change in protein/flotillin-1 ratio was calculated (the protein/flotillin-1 ratio for resveratrol-treated tissues was set in relation to preparations from control tissues (*n* = 6, *n* = 4 for pNHE3 Ser605 Ileum)).

Protein	Jejunum	Ileum
SGLT1	1.41 ± 0.87	0.82 ± 0.45
PEPT1	1.17 ± 0.76	2.27 ± 2.21
NHE3	1.76 ± 2.12	1.06 ± 0.96
pNHE3 Ser552	1.44 ± 1.57	1.35 ± 0.93
pNHE3 Ser605	2.18 ± 1.80	0.62 ± 0.28
